# Supplementary material for: PM2.5 toxin benzo[a]pyrene induces life-limiting inflammation and oxidative stress in the airway by up-regulation of TRPC6 and inactivation of β2AR/CFTR signaling
Source: bioRxiv. 2026 Apr 24:2026.04.21.719931. Preprint. [Version 1] doi: 10.64898/2026.04.21.719931 (PMC13131649; doi:10.64898/2026.04.21.719931)
Supplement: Supplement 1 [file NIHPP2026.04.21.719931v1-supplement-1.pdf]

bioRxiv preprint doi: https://doi.org/10.64898/2026.04.21.719931; this version posted April 24, 2026. The copyright holder for this preprint (which was not certified by peer review) is the author/funder. This article is a US Government work. It is not subject to copyright under 17 USC 105 and is also made available for use under a CC0 license.

SUPPLEMENTAL DATA

PM2.5 toxin benzo[a]pyrene induces inflammation and oxidative stress in the airway by up-regulation of TRPC6 and inactivation of β2AR/CFTR signaling

Hung Caohuy [1], Ognoon Mungunsukh [1,2,], Tinghua Chen [1], Qingfeng Yang [3], Thalia Dib [1], Bette S. Pollard [4], Naheed Fatima [1], Thomas Flagg [1], Dharmendra K. Soni [1,5], Roopa Biswas [1], William Rittase [1], Oliver J L'Esperance [1], Sharon Juliano [1], and Harvey B. Pollard [1]

1. Department of Anatomy, Physiology and Genetics (APG), Uniformed Services University School of Medicine, Uniformed Services University of the Health Sciences, Bethesda, MD 20814.

2. Center for Military Precision Health (CMPH), Uniformed Services University of the Health Sciences, Bethesda, MD 20814.

3. Center for the Study of Traumatic Stress (CSTS), Uniformed Services University of the Health Sciences, Bethesda, MD 20814.

4. Silver Pharmaceuticals, Rockville, MD

5. Current Address: Amity Institute of Biotechnology, Amity University, Haryana, Gurugram, India

bioRxiv preprint doi: https://doi.org/10.64898/2026.04.21.719931; this version posted April 24, 2026. The copyright holder for this preprint (which was not certified by peer review) is the author/funder. This article is a US Government work. It is not subject to copyright under 17 USC 105 and is also made available for use under a CC0 license.

Supplemental Figure S1

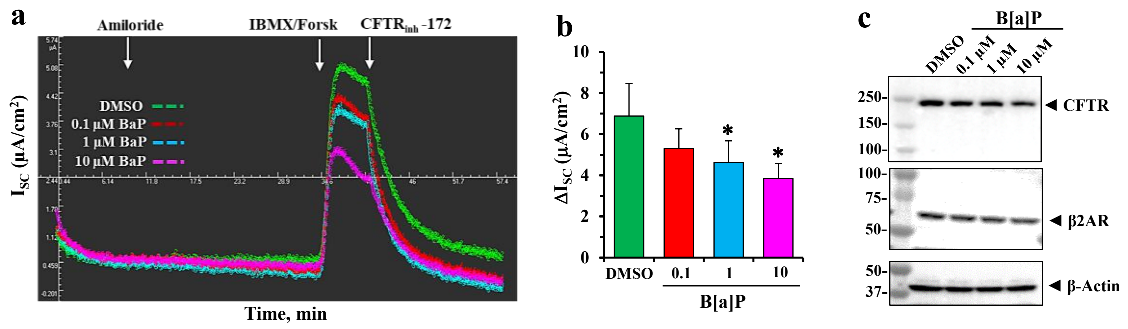

Supplemental Figure S1: Effects of Benzo[a]Pyrene (B[a]P) on functional expression of CFTR in polarized 16HBE14o cells. Both apical and basolateral sides of polarized 16HBE14o- cells (ALI for 8-10 days) were incubated with different concentrations of B[a]P. Treated media from the apical sides were removed after 6-hr incubation, and cell treatment continued further for 18 hrs under ALI conditions. (**a**) Representative I_{sc} tracings of CFTR-dependent short-circuit currents (I_{sc}) measured in Ussing chambers as the changes in response to Amiloride, IBMX/Forskolin, and CFTRinh-172. (b) Summary of changes in I_{sc} is shown. Data represent the mean of three independent experiments, and each experiment was performed in duplication (n = 3). Statistical *p* values were determined with a one-way ANOVA, followed by the Tukey post-hoc test comparing the B[a]P treatments to the DMSO control; * $p < 0.05$; ** $p < 0.01$. (**c**) Representative Western blot images of β2AR and CFTR expression levels in treated cells after Ussing chamber analyses in (**a**).

bioRxiv preprint doi: https://doi.org/10.64898/2026.04.21.719931; this version posted April 24, 2026. The copyright holder for this preprint (which was not certified by peer review) is the author/funder. This article is a US Government work. It is not subject to copyright under 17 USC 105 and is also made available for use under a CC0 license.

Supplemental Figure S2

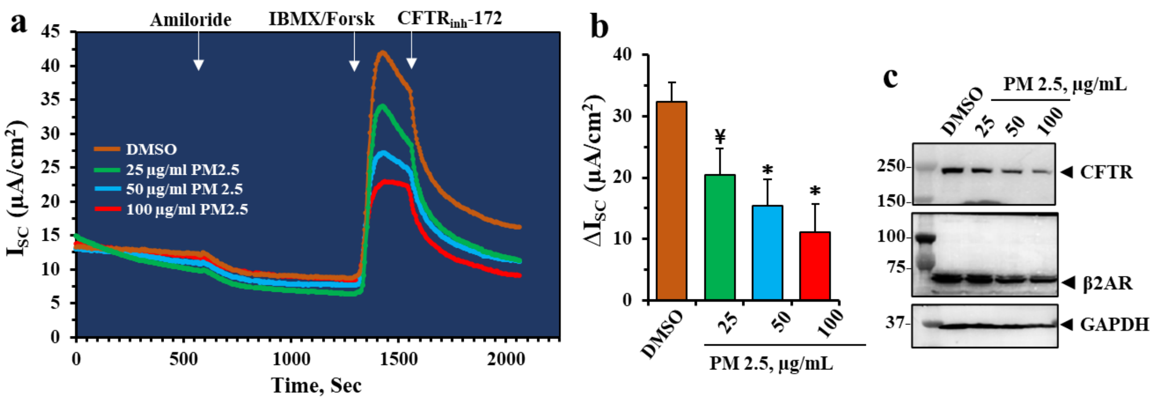

Supplemental Figure S2: Effects of PM 2.5 on functional expression of CFTR in polarized 16HBE14o cells. Both apical and basolateral sides of polarized 16HBE14o- cells (ALI for 8-10 days) were incubated with different concentrations of PM 2.5. Treated media from the apical sides were removed after 6-hr incubation, and cell treatment continued further for 18 hrs under ALI conditions. (**a**) Representative I_{sc} tracings of CFTR-dependent short-circuit currents (I_{sc}) measured in Ussing chambers as the changes in response to Amiloride, IBMX/Forskolin, and CFTRinh-172. (b) Summary of changes in I_{sc} is shown. Data represent the mean of three independent experiments, and each experiment was performed in duplication (n = 6). Statistical *p* values were determined with a one-way ANOVA, followed by the Tukey post-hoc test comparing the B[a]P treatments to the DMSO control; ¥, $p < 0.05$; ∗, $p < 0.01$. (**c**) Representative Western blot images of β2AR and CFTR expression levels in treated cells after Ussing chamber analyses in (**a**). All Western analyses represent the results of three independent experiments, and β-actin was used for equal loading of protein.

bioRxiv preprint doi: https://doi.org/10.64898/2026.04.21.719931; this version posted April 24, 2026. The copyright holder for this preprint (which was not certified by peer review) is the author/funder. This article is a US Government work. It is not subject to copyright under 17 USC 105 and is also made available for use under a CC0 license.

Supplemental Figure S3

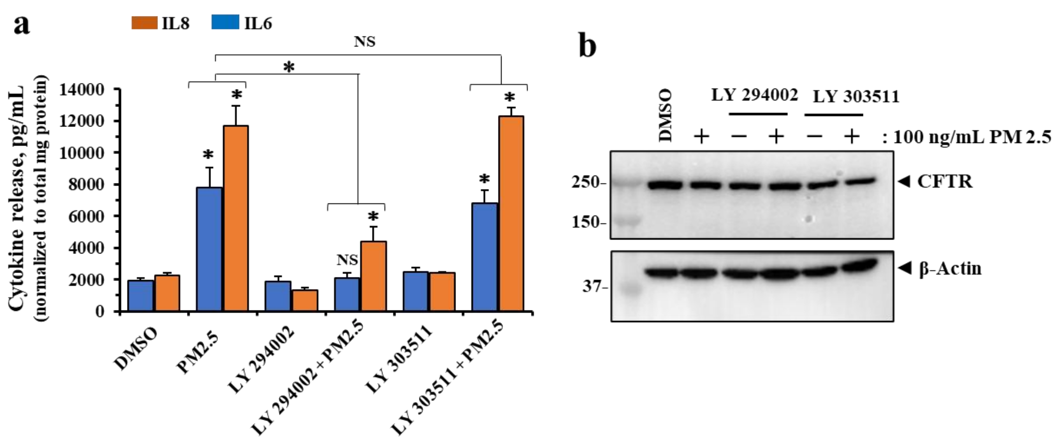

Supplemental Figure S3. PM 2.5 induces cytokine secretion via the PI3K signaling pathway in polarized 16HBE14o- cells. ALI cells were treated with DMSO, 100 ng/mL PM 2.5, 10 µM LY 294002 ± PM 2.5, or 10 µM LY 303511 ± PM 2.5 for 24 hrs. (**a**) IL-6 and IL-8 from the basolateral sides were determined using the DuoSet® ELISA kits. Data are presented as the mean ± SD (n=3). Statistical *p* values were determined with a one-way ANOVA, followed by the Tukey post-hoc test comparing all treatments to the DMSO control, or PM 2.5 to PM 2.5 ± LY 294002 or LY 303511; ∗, $p < 0.01$. (**b**) Representative Western blot images showing CFTR and β-actin expression. β-actin was used to ensure equal loading of protein. All data were obtained from three independent experiments, and each experiment was analyzed in duplication.

bioRxiv preprint doi: https://doi.org/10.64898/2026.04.21.719931; this version posted April 24, 2026. The copyright holder for this preprint (which was not certified by peer review) is the author/funder. This article is a US Government work. It is not subject to copyright under 17 USC 105 and is also made available for use under a CC0 license.

Supplemental Figure S4

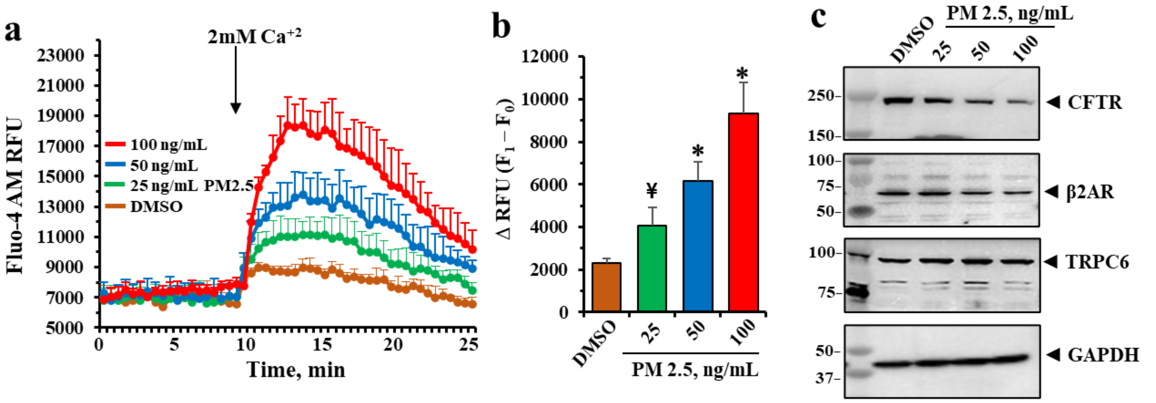

Supplemental Figure S4. PM 2.5 induces calcium influx in 16HBE14o- cells. (**a**) After 24 hr treatment, fluorescence intensity traces obtained using the fluo-4 AM when DMSO- or PM 2.5-treated 16HBE14o- cells were stimulated with assay buffer containing 2 mM Ca^{+2}. Each trace is the mean of three independent experiments with standard deviations (vertical bars). (**b**) Corresponding histograms summarize the mean area under the curve (AUC) after stimulation by 2 mM Ca^{+2}. (**c**) Representative Western blot images of CFTR, β2AR and TRPC6 expression levels in treated cell in (**a**). All data were obtained from four independent experiments. Statistical *p* values were determined with a one-way ANOVA, followed by the Tukey post-hoc test comparing the PM 2.5 treatments to the DMSO control; ¥, $p < 0.05$; ∗, $p < 0.01$.

bioRxiv preprint doi: https://doi.org/10.64898/2026.04.21.719931; this version posted April 24, 2026. The copyright holder for this preprint (which was not certified by peer review) is the author/funder. This article is a US Government work. It is not subject to copyright under 17 USC 105 and is also made available for use under a CC0 license.

Supplemental Figure S5

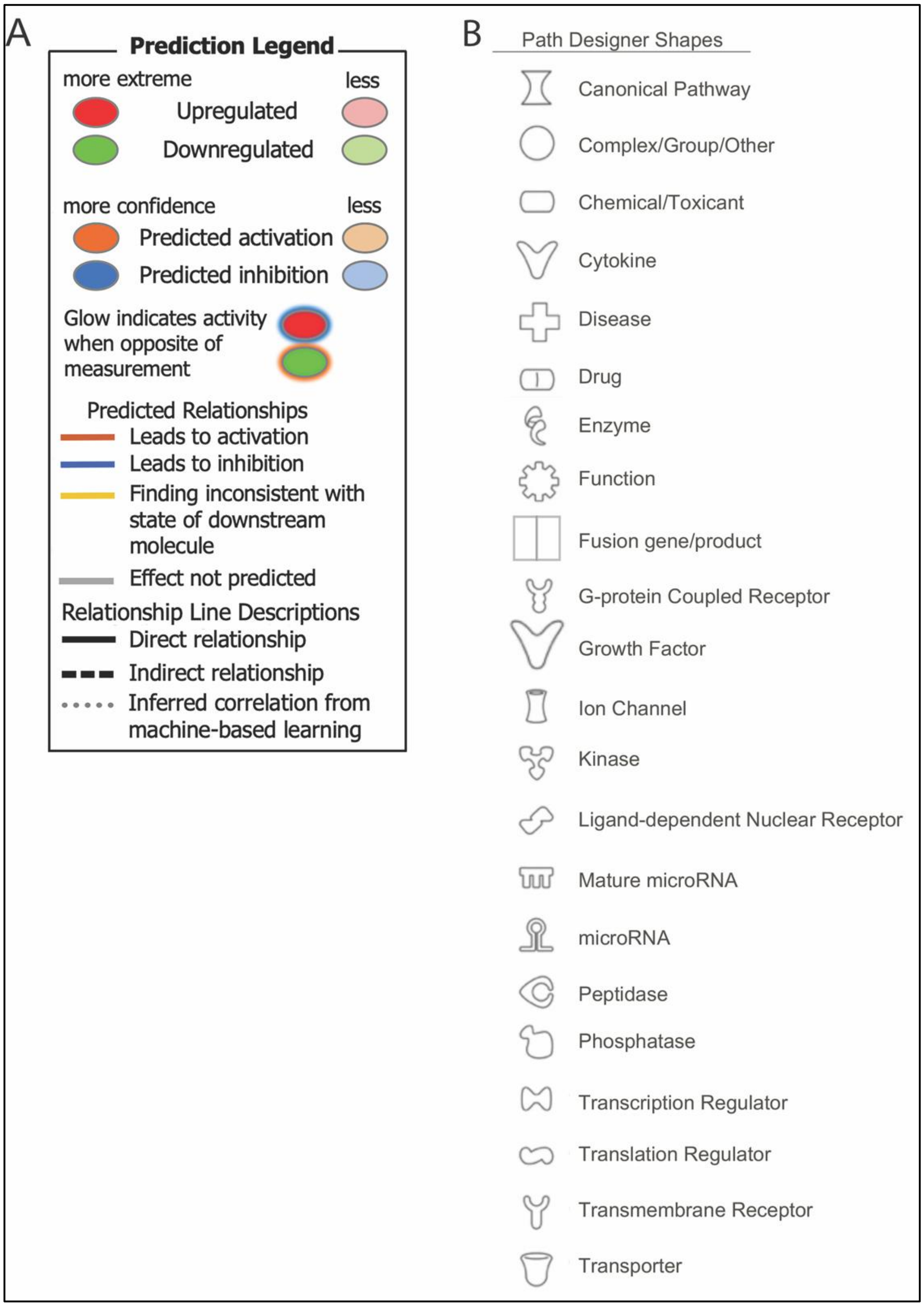

Prediction Legend for Ingenuity Pathway Analysis, Qiagen.com
